# Case Report: From critical trauma to functional recovery: a novel surgical and hand-rearing strategy in a neonatal Hamadryas baboon (*Papio hamadryas*)

**DOI:** 10.3389/fvets.2026.1885581

**Published:** 2026-08-21

**Authors:** Prashant Shivaji Gurav, Anand Rajshekhar Dadke, Swapnil Ashokrao Jadhav, Rucha Gaurav Shrivastav, Mahesh Babasaheb Pawar

**Affiliations:** Department of Wildlife Health and Management, Greens Zoological Rescue and Rehabilitation Centre, Jamnagar, Gujarat, India

**Keywords:** anorectal reconstruction, caudectomy, hand-rearing, infant-directed aggression, neonatal surgery, *Papio hamadryas*

## Abstract

Infanticide and infant-directed aggression are recognized behaviors in non-human primates and can occasionally lead to severe neonatal trauma. This report describes the successful management of a life-threatening anorectal and sacro-coccygeal injury in a neonatal Hamadryas baboon (*Papio hamadryas*) following an aggressive encounter with an adult male. The neonate presented in critical condition with profuse hemorrhage, sacro-coccygeal dislocation, and extensive damage to the perineal and anorectal tissues, resulting in hypovolemia and hemodynamic instability. After initial stabilization, emergency surgery was performed under isoflurane anesthesia. Owing to complete dislocation and non-viability of the distal tail, a therapeutic caudectomy was carried out, accompanied by anorectal reconstruction through debridement, mobilization of the rectal mucosa, and restoration of anorectal continuity. A temporary rectal stent was placed to maintain patency during healing. Post-operative management included antimicrobial therapy, analgesia, thermal support, and intensive monitoring. Considering the risk of recurrent aggression, the infant was subsequently hand-reared under controlled environmental conditions with structured nutritional support. Steady weight gain from 602 g at birth to 1.5 kg at 15 weeks, along with the return of normal fecal passage, indicated satisfactory functional recovery. This case highlights the importance of prompt stabilization, meticulous surgical intervention, and dedicated post-operative care in achieving favorable outcomes following severe anorectal trauma in neonatal non-human primates. It also contributes to the limited body of literature describing caudectomy and anorectal reconstruction in this age group.

## Introduction

Hamadryas baboons (*Papio hamadryas*) live in remarkably complex social systems made up of one-male units, clans, and larger bands. These structures are usually stable and well-organized, but episodes of male-driven aggression, including infanticide, have been reported in both wild and captive settings (1, 2). In many primate species, infanticide is often explained by the sexual selection hypothesis—where a male may eliminate an unrelated infant to bring the female back into reproductive readiness sooner (1). Despite their generally protective role toward females and infants, male Hamadryas baboons can display infant-directed aggression under certain circumstances. Situations involving social instability, competition, or uncertainty of paternity can trigger such behavior. These risks may be even more pronounced in facility-managed environments, where natural social dynamics are inevitably altered (3, 4). In managed settings, factors like limited space, changes in group composition, and constant proximity can increase stress and tension within the group—even among individuals that previously appeared compatible (2, 5). The time around birth is particularly sensitive, as newborns are especially vulnerable during this period (5). Alongside traumatic threats, neonatal survival can also be challenged by congenital conditions such as anorectal malformations, which demand timely medical attention. This case describes a rare, unfortunate and successful therapeutic management of male-induced trauma in a newborn Hamadryas baboon in a facility-managed setting. The infant sustained a sacro-coccygeal dislocation along with severe anorectal injury. Prompt recognition of the severity of the condition and immediate surgical intervention played a crucial role in successfully managing the case, highlighting both the challenges and the importance of vigilant care in such environments.

## Case description

A small facility-managed group of Hamadryas baboons (*Papio hamadryas*)—one adult male, one subadult male, and two adult females—was housed at the Greens Zoological Rescue and Rehabilitation Center in Jamnagar. They were kept in a well-planned enclosure that included a comfortable, air-conditioned indoor space (25 ft × 20 ft) connected to a larger outdoor area (40 ft × 25 ft x 30 ft). The outdoor section was partly covered and secured with strong stainless-steel fencing, allowing fresh air while ensuring the animals safety. Care had been taken to make the enclosure as natural and engaging as possible. It was equipped with wooden perches at different heights, raised platforms, and rope systems that allowed the baboons to climb, move, and explore—just as they would in the wild. Hiding boxes were also available, giving them a place to retreat and feel secure when needed. The animals were fed three times a day, with meals served in stainless steel bowls. Their diet was varied and nutritious, including fresh browse, vegetables, leafy greens, seasonal fruits, and nuts. To keep them mentally stimulated and encourage natural foraging behavior, enrichment foods like eggs, sweet corn, chicken, sugarcane, and coconut were offered on a rotating basis. Before the incident, the group appeared healthy and well-adjusted, with no signs of illness or social conflict. One of the adult females was in advanced pregnancy and continued to interact normally and calmly with both males. She gave birth in the morning, and at first, everything seemed normal. The mother showed normal maternal behavior by nursing and grooming the infant. However, shortly after the birth, the adult male approached them and tried to take the baby. The mother resisted and attempted to protect her infant, but the situation quickly escalated. The male became aggressive and bit the newborn, causing severe injuries. The infant was immediately rescued by the caretakers and rushed for emergency veterinary treatment, marking a sudden and distressing turn in what had initially appeared to be a normal, healthy birth.

## Clinical findings

The newborn was in a critical condition, with heavy bleeding from the tail region and a dislocation at the sacro-coccygeal joint. There was severe damage to the soft tissues around the perineal and anorectal areas, and the infant was clearly in significant pain and distress. The patient was considered to be in a life-threatening condition based on objective physiological assessment, including heart rate, respiratory rate, peripheral oxygen saturation (SpO_2_), capillary refill time, and body temperature together with ongoing hemorrhage and extensive perineal trauma. In addition, the severity of the traumatic injury raised concerns regarding the potential development of systemic sepsis.

## Diagnosis

The injuries were consistent with a traumatic bite inflicted during aggressive interaction with another member of the group. The newborn presented with a complete sacro-coccygeal dislocation associated with extensive anorectal soft tissue injury, including damage to the surrounding muscles and supporting perineal structures. The severity of the trauma caused significant distortion of the normal anatomy, extensive tissue loss, and compromised anorectal function, warranting immediate surgical intervention. Radiographs, advanced imaging, and initial preoperative photographs were not obtained because the patient was a newborn who was actively bleeding, contaminated, and at risk of hypovolemic shock. In this emergency setting, delaying surgical intervention for imaging or photographic documentation was deemed clinically inappropriate. Intraoperative and post-operative images were obtained once the neonate had been adequately stabilized and are included as figures.

## Therapeutic and surgical management

After initial stabilization, the neonate was taken for emergency surgery under general anesthesia using isoflurane. The anesthetic approach was dictated by emergency physiology rather than convenience. Neonates have limited physiological reserves, including small circulating blood volume, immature thermoregulation, limited glycogen stores, immature hepatic and renal function, and reduced ability to compensate for hypoxemia or hemorrhage (6–8). In the present case, the patient was actively bleeding and contaminated; therefore, rapid initiation, controllability, and recovery profile were prioritized.

Isoflurane in oxygen was selected because it offered several practical advantages in this emergency setting. It could be administered immediately without delaying surgery for intravenous catheterization or complex injectable drug calculations. The depth of anesthesia could be adjusted rapidly in response to surgical stimulation, and recovery was expected to occur relatively promptly after discontinuation. In addition, isoflurane has low systemic metabolism and is eliminated predominantly through pulmonary exhalation, reducing reliance on immature hepatic metabolic pathways (9–11). Peer-reviewed non-human-primate anesthesia literature also supports the clinical use of inhalational agents, including isoflurane, as well as injectable agents such as ketamine and propofol in macaques (12, 13). In this newborn, which was already restrained for emergency care and required immediate surgical intervention, inhalational induction and maintenance represented the most practical and least cumulative option.

The main alternatives had disadvantages in this specific case. Ketamine combined with a benzodiazepine, such as diazepam or midazolam, could have provided restraint and some cardiovascular support; however, injectable anesthetic duration and recovery may be less predictable in neonates because of developmental pharmacokinetic variability and immature drug clearance (6, 12, 13). Ketamine–alpha-2 agonist combinations are used in non-human primates, but alpha-2 agonists are associated with bradycardia and reduced cardiac output, which was undesirable in a neonate with suspected hypovolemia (12, 14). An opioid-heavy balanced anesthetic technique could have improved analgesia, but it would also increase the risk of respiratory depression and might require more intensive ventilatory support (15). Propofol or alfaxalone would have required reliable intravenous access; propofol, in particular, has been associated with dose-dependent hypotension in neonates (16). Sevoflurane would have been a reasonable alternative because of its low blood solubility, rapid uptake, and rapid elimination, but the available isoflurane system was familiar, immediately functional, and adequate for a short emergency procedure (17, 18). Thus, the decision reflects risk minimization under real emergency conditions, not a claim that isoflurane is universally superior.

**Figure 1:**
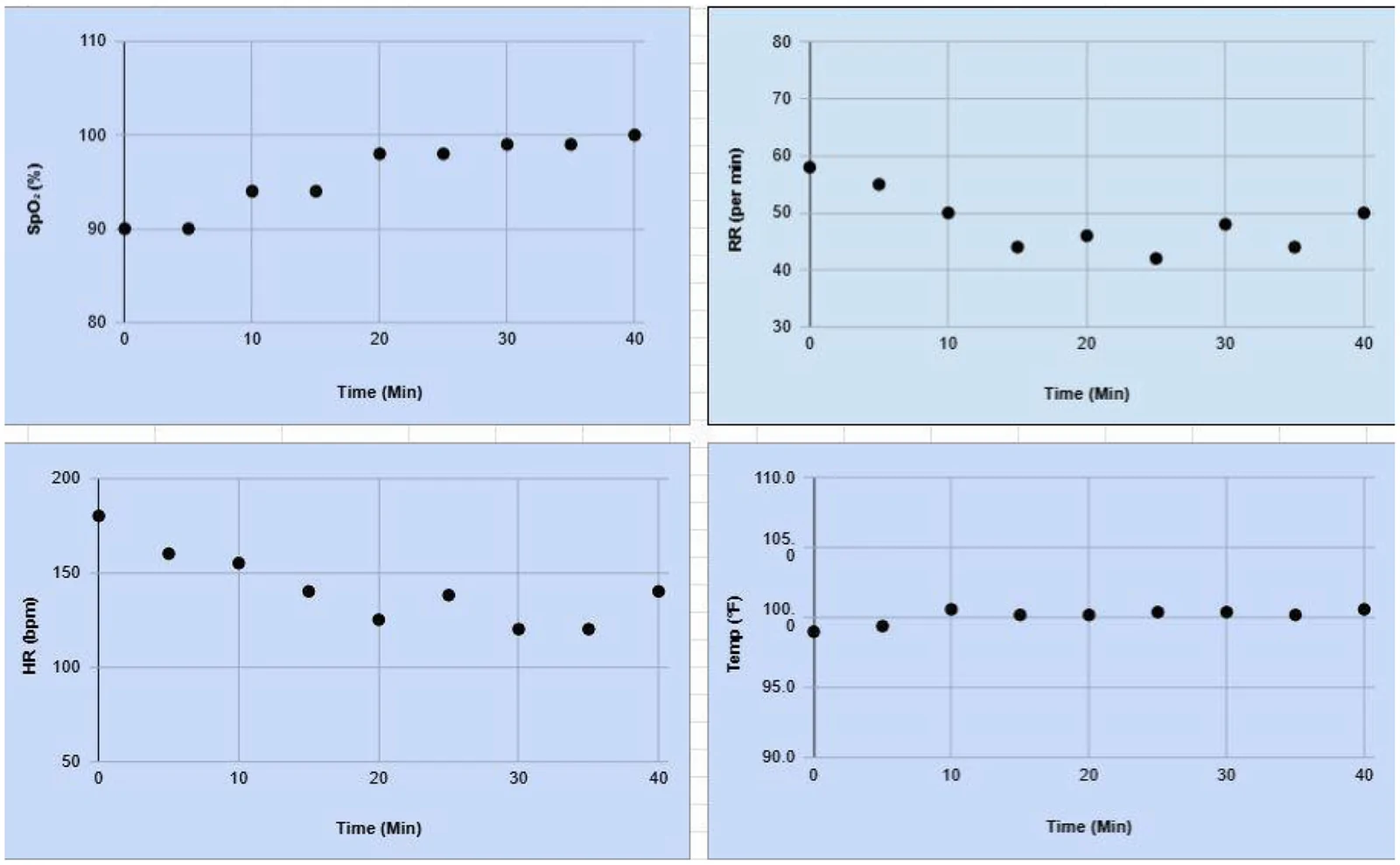
Intraoperative monitoring of vital parameters during a 40-min surgical procedure in a neonatal Hamadryas baboon (*Papio hamadryas*).

Considering the delicate condition of such a young patient, extra care was taken to closely monitor breathing and maintain body temperature throughout the procedure. The neonate was monitored continuously during the 40-min procedure using a multiparameter monitor. Intraoperative assessment included HR, RR, SpO_2_, and RT (Figure 1). On examination, the tail was found to be completely dislocated at the sacro-coccygeal joint, and the distal portion showed no response, clearly indicating loss of sensation and non-viability. Given this, a caudectomy was carried out. The incision was made just proximal to the damaged area, ensuring a healthy tissue margin. The affected sacro-coccygeal vertebrae and all non-viable tissues were carefully removed, bleeding was controlled by ligation, and the area was gently irrigated with sterile saline. The wound was then closed in layers using absorbable synthetic suture material (polyglactin 910, 2-0) for the musculature and nylon 2-0 for the skin, following a standard and secure closure technique.

**Figure 2:**
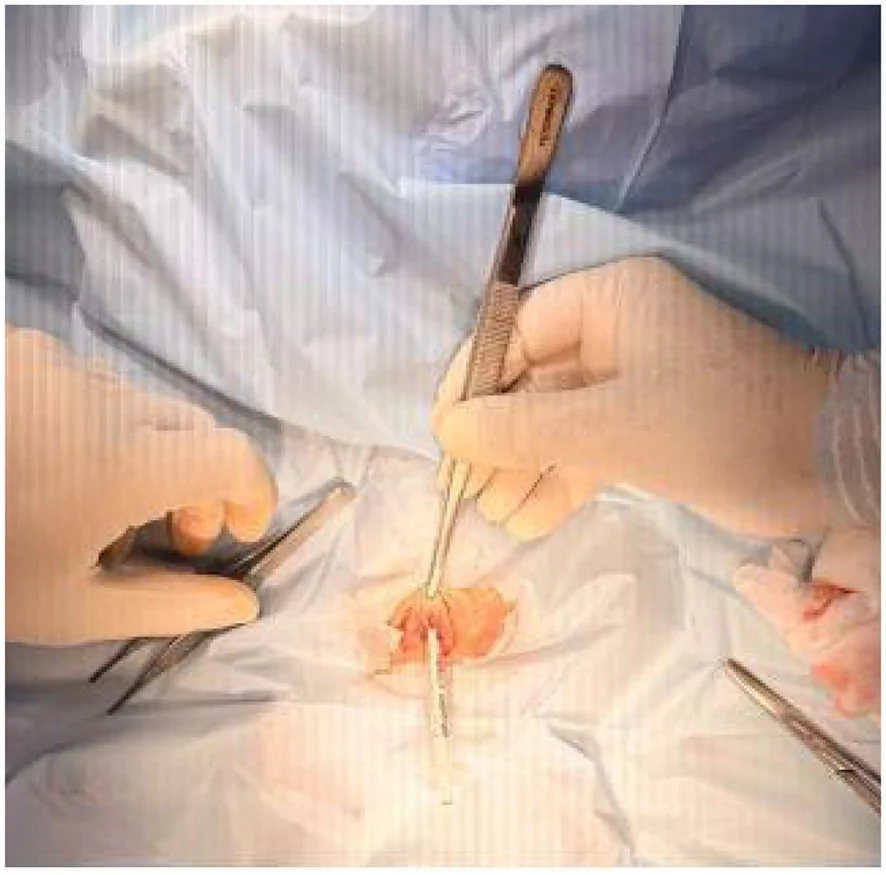
Intraoperative view showing caudectomy and anorectal reconstruction in the neonatal Hamadryas baboon.

**Figure 3:**
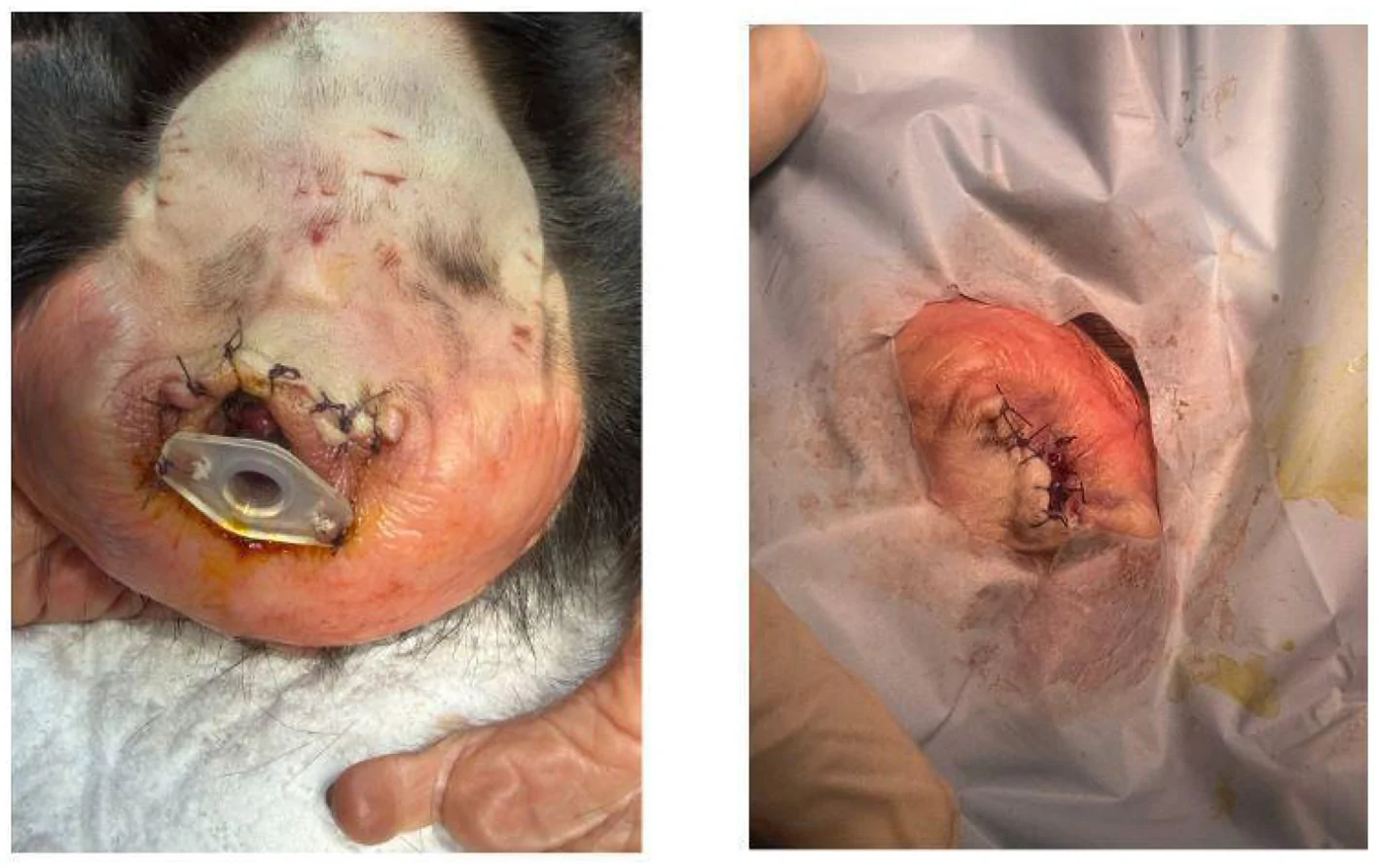
Immediate post-operative appearance following successful surgical reconstruction.

Attention was then turned to the perineal region, where a large, irregular lacerated wound extending up to the base of the tail was observed, along with abnormal fecal discharge. The area was handled with great care—thoroughly cleaned, and all accumulated fecal material was gently removed. After adequate debridement, the cavity was flushed with diluted povidone-iodine solution to minimize contamination. The rectal mucosa was then carefully identified, mobilized, and brought outward. Reconstruction of the anorectal opening was performed with precision, suturing the mucosa to the surrounding skin in an everted manner to recreate a functional and anatomically appropriate opening. Its alignment and patency were checked intraoperatively to ensure that normal defecation would be possible post-surgery (Figure 2). To further safeguard the repair and reduce the chances of complications such as fecal contamination, narrowing of the opening, or wound breakdown, a temporary rectal stent was placed. This was fashioned from a modified 1 ml BD syringe, with its edges smoothened to avoid irritation (Figure 3). It was gently inserted into the rectum to help maintain lumen patency and secured in place with sutures for stability.

**Figure 4:**
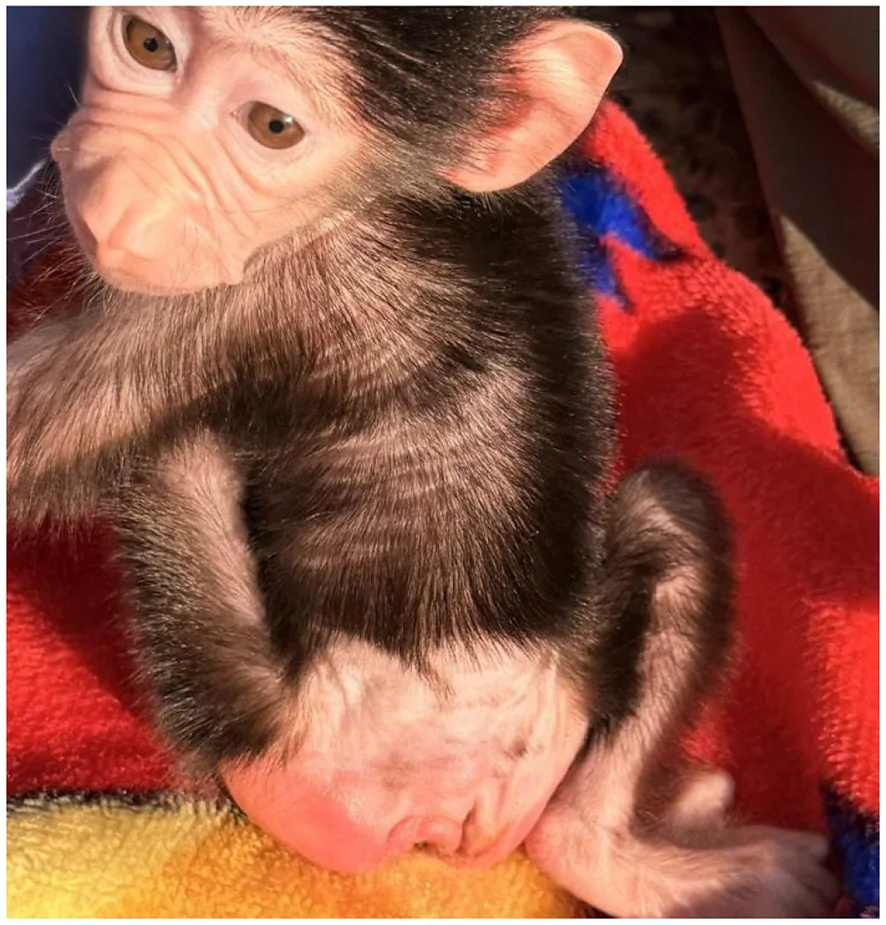
Early post-operative recovery with stable healing at day 10.

Following the procedure, the neonate was given attentive post-operative care to support recovery. Broad-spectrum antibiotic amoxicillin-clavulanate (13 mg/kg) was administered orally BID for seven days to prevent infection, along with meloxicam (0.2 mg/kg) to manage pain for 3 days. Particular attention was paid to maintaining body temperature and fluid balance, both of which are critical in neonates. Fluids were administered intravenously as a constant rate infusion (CRI) at 20 mL/kg/hour. Meloxicam was given intramuscularly before surgery. The first dose of antibiotic, amoxicillin–clavulanic acid, was administered parenterally, after which it was continued orally. The surgical site was cleaned regularly and closely observed for any signs of infection or wound complications. Recovery was monitored not only by the healing of the wound but also by observing the passage of feces through the reconstructed opening and the overall activity and responsiveness of the neonate. After 10 days, the sutures and rectal stent were removed, and the healing was found to be satisfactory, with restoration of normal function and a smooth recovery overall (Figure 4).

## Hand-rearing, growth monitoring, and nutritional management

Following surgical intervention, and considering the severity of trauma, the neonate was placed under intensive hand-rearing management to support survival and recovery. The infant was maintained in a carefully controlled environment with adequate thermal support to prevent hypothermia, which is critical in neonatal care (19). Assisted feeding was initiated using a suitable milk substitute formulated for neonatal primates, administered at regular intervals. Strict hygiene protocols were followed during feeding and handling to minimize the risk of infection. Continuous monitoring was carried out, focusing on body temperature, feeding response, hydration status, defecation through the reconstructed anal opening, and overall activity and reflexes. This comprehensive supportive care played a vital role in stabilizing the neonate and promoting post-operative recovery.

Baseline morphometric parameters were recorded on the first day of life to establish reference values for growth monitoring. The neonate weighed 602 g and showed morphometric measurements consistent with the expected range for a neonatal Hamadryas baboon. Detailed measurements are presented in Table 1.

**Table 1:** Growth monitoring: morphometric parameters.

Morphometric parameters	Day old	4 months
Body weight	602 g	1.5 kg
Head width	5.6 cm	7 cm
Head length	7.2 cm	11 cm
Head height	Not recorded	7 cm
Head circumference	21 cm	26 cm
Crown to rump length	15 cm	27 cm
Chest circumference	18 cm	22 cm
Arm length	Not recorded	25 cm
Hand length	Not recorded	6 cm
Leg length	Not recorded	28 cm
Foot length	6.7	9 cm

Growth was monitored regularly using body weight as a primary indicator of health and nutritional status. The neonate demonstrated a steady and progressive increase in body weight throughout the monitoring period (From day-old to 15th week), indicating adequate nutritional support and a favorable post-operative recovery trajectory.

The feeding protocol was carefully structured to meet the neonate's nutritional needs. A commercially available infant formula (NanPro milk powder^1^™) was used, prepared by dissolving one spoon of milk powder in 30 ml of lukewarm water. During the first week, the total daily intake was maintained at approximately 15–20% of body weight, divided into 12 feedings given at roughly 2-h intervals. As the neonate grew, the feeding frequency was gradually reduced to eight times daily in the second week, 6 times per day in the third week, and 5 times per day from the fourth week onwards.

Beginning in the eighth week, supplementary feeding was gradually introduced to support nutritional diversification and developmental adaptation. This included mixed fruits and vegetables provided three times daily, along with boiled chicken and boiled eggs once daily. This transition helped the neonate adjust to a more varied diet while continuing to support growth.

Housing and environmental management were also carefully planned to support recovery and development. For the first 2 weeks, the neonate was maintained in a curadle™ with controlled thermal support. Daily exposure to early morning sunlight for 30 min to 1 h was provided to promote overall health. Orally multivitamins and probiotics were provided as a supplement. From the third week onwards, the neonate was shifted to a small stainless steel suspended indoor enclosure. By the fourth week, controlled access to an outdoor enclosure during the daytime was introduced, while nighttime housing remained indoors. This gradual environmental exposure facilitated acclimatization, encouraged physical activity, and supported normal behavioral development.

**Figure 5:**
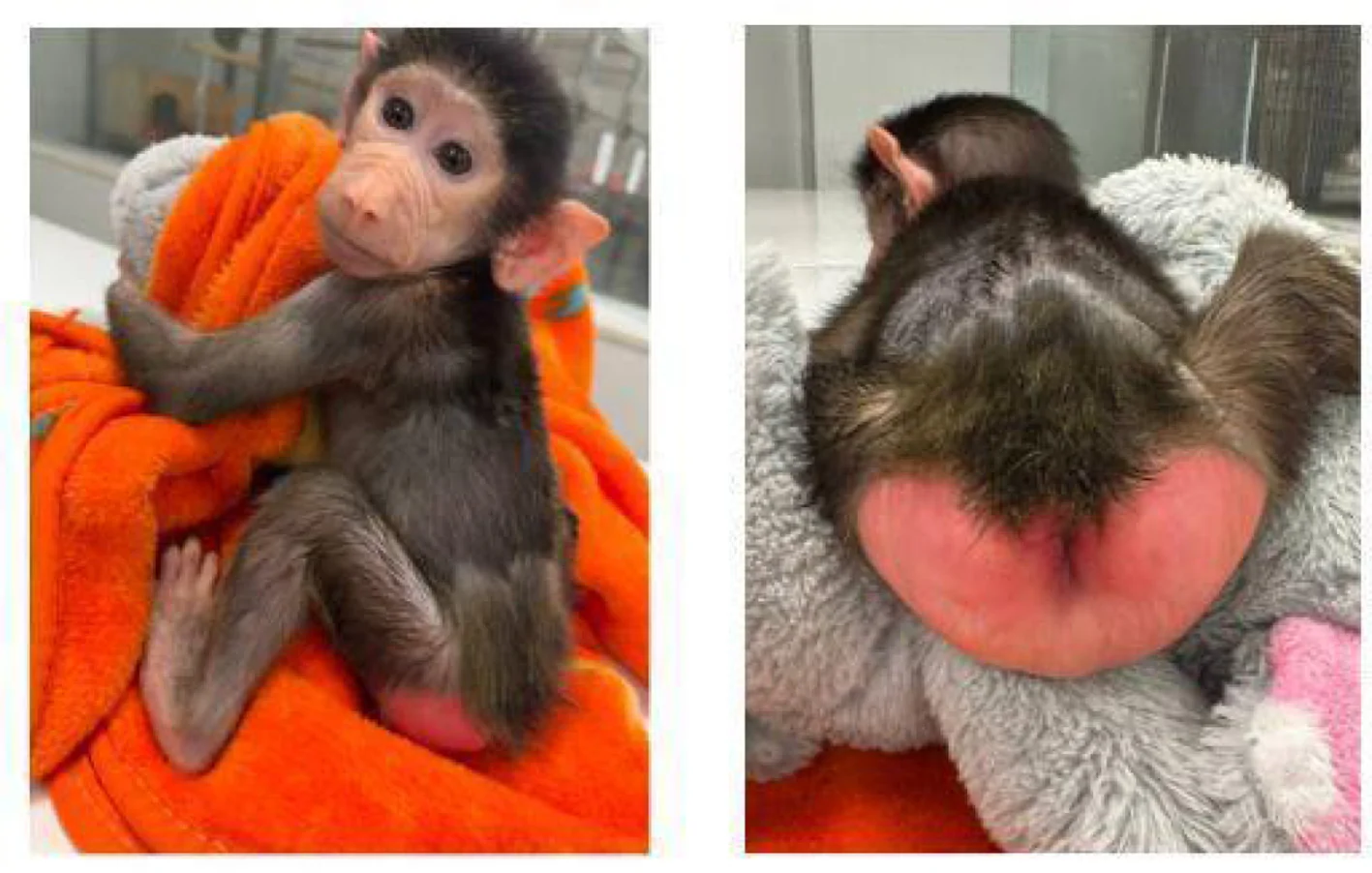
Four-month post-operative outcome demonstrating complete healing and functional recovery.

Overall, the neonate responded well to hand-rearing management. It demonstrated a good feeding response, steady weight gain, normal activity levels, and successful defecation through the reconstructed anal opening. These outcomes indicated effective post-operative care and a favorable recovery (Figure 5). Clinical progress was assessed based on locomotor activity, gastrointestinal function, wound healing, feeding response, and behavioral observations. A summary of the major clinical findings recorded during follow-up is presented in Table 2.

**Table 2:** Serial clinical evaluations during post-operative recovery and hand-rearing.

Time point	Locomotion/activity	Gastrointestinal status	Wound healing	Behavioral observations
Day 1	Limited activity; responsive to handling	No Feces Passed	Surgical site clean; mild post-operative erythema	neonatal reflexes intact with comparative less milk intake
Day 3	Increased spontaneous movement and activity	Regular fecal passage; no signs of obstruction	Sutures intact and no evidence of infection	Good feeding response
Day 7	Active movement within Intensive ICU	Normal defecation maintained	Progressive healing with minimal soiling	Alert and responsive to caregivers
Day 10	Normal neonatal mobility	Normal fecal consistency and frequency	healing satisfactory and skin sutures removed	Adapted well to hand-rearing
Week 4	Active climbing and exploratory behavior	Normal gastrointestinal function	Complete external wound healing	Normal interaction with caregivers with weight gain
Week 8	Age-appropriate locomotor activity	Continued normal defecation	No post-operative complications observed	Successfully transitioned to supplementary feeding
Week 15	Normal activity and coordination	Normal gastrointestinal function maintained	Functional recovery maintained	No Significant movement difficulty

## Outcome

**Figure 6:**
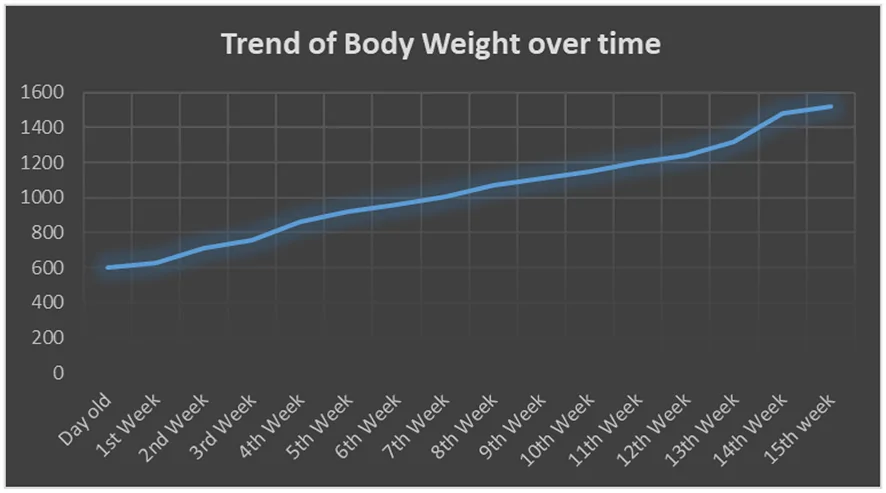
Fifteen-week body weight trajectory demonstrating sustained neonatal recovery.

Post-operatively, the neonate recovered uneventfully. Hemostasis was achieved, the surgical site healed without infection, dehiscence, stenosis, or other perineal complications, and normal defecation through the reconstructed anal opening was restored. The rectal stent was removed on day 10 without complications. With hand-rearing and nutritional support, the infant remained active, fed well, and gained weight steadily from 602 g at birth to approximately 1.5 kg by week 15, indicating complete functional recovery (Figure 6). Overall, this case illustrates how timely surgical intervention, combined with attentive post-operative care and dedicated nutritional management, can lead to a successful outcome even in severe neonatal trauma.

## Discussion

Infanticide and infant-directed aggression are well-recognized behaviors in non-human primates and are often linked to male reproductive strategies, particularly under the sexual selection hypothesis (1, 3). Although Hamadryas baboons are generally known for their well-organized and stable social structures, such aggressive incidents, though uncommon, can still occur—especially in situations involving social stress, instability, or uncertainty of paternity (2, 4).

In captive or facility-managed settings, natural social dynamics are often altered. Factors such as limited space, close proximity, and changes in group composition can increase stress levels and may contribute to unexpected or heightened aggression (5). The period around birth is particularly sensitive, as newborns are extremely vulnerable, and even strong maternal care may not always be enough to protect them. This case supports earlier observations that the immediate postnatal period carries a higher risk of infant-directed aggression, highlighting the need for careful monitoring and proactive management during this time.

From a clinical standpoint, this case illustrates how severe and life-threatening such injuries can be in neonatal primates. It also emphasizes the importance of acting quickly. The successful management of this case demonstrates that even complex surgical procedures, such as caudectomy and anorectal reconstruction, can be safely performed in very young and fragile patients when carried out with proper anesthetic care, surgical precision, and attentive post-operative support. The use of a temporary rectal stent was particularly helpful in maintaining patency and reducing the risk of complications such as narrowing or contamination.

In companion animals like dogs and cats, partial caudectomy is generally considered a safe and well-tolerated procedure, with little impact on normal function. In most cases, pet owners do not notice any obvious changes in their animal's behavior after surgery (20). However, when it comes to primates, little is currently known about how this procedure might affect their behavior or overall physiology. This gap in understanding highlights the need for more research, particularly in free-ranging primates where the tail may play a more important role in movement, balance, and social interactions.

The extent of tissue injury in the present case raised concerns regarding potential neurological involvement, particularly because the sacro-coccygeal region contributes to the innervation of the perineal musculature and continence mechanisms. However, no gross damage to the anal sphincter complex was observed during surgical exploration. Although a detailed neurological assessment was not performed, restoration of normal fecal passage, maintenance of anorectal patency, and the absence of clinically evident continence deficits throughout the observation period indicated satisfactory short-term functional recovery. Nevertheless, the possibility of long-term sequelae associated with sacro-coccygeal trauma cannot be completely excluded, and continued follow-up is warranted to better understand the long-term functional outcomes of such injuries in non-human primates.

This case has certain limitations that should be acknowledged. Because the neonate presented with severe hemorrhage, extensive perineal trauma, and clinical evidence of hypovolemia, immediate stabilization and surgical intervention were prioritized. As a result, preoperative photographic documentation and diagnostic imaging could not be obtained before surgery. Although this limited the objective documentation of the patient's initial condition, delaying treatment to obtain additional records would not have been in the patient's best interest. Future reports of similar emergency cases would benefit from including detailed physiological measurements, diagnostic imaging, and photographic documentation whenever the patient's clinical condition allows. Such imaging could have enabled a more detailed assessment of the sacro-coccygeal injury, the extent of soft tissue damage, and any potential neurological involvement, in addition to providing objective post-operative documentation.

Species-specific treatment guidelines for neonatal Hamadryas baboons are currently unavailable, and clinical management in such cases often depends on principles derived from neonatal veterinary medicine, non-human primate practice, and pediatric critical care. In the present case, antimicrobial, analgesic, and fluid therapy protocols were chosen to minimize the risk of wound infection, provide adequate pain management, and maintain cardiovascular stability. The favorable short-term outcome observed suggests that these interventions can serve as a practical therapeutic approach for managing severe anorectal trauma in neonatal non-human primates. Nevertheless, additional studies and clinical reports are needed to develop evidence-based treatment recommendations for this age group and species.

In the present case, the decision to perform caudectomy was not elective but clearly necessary. The tail had sustained dislocation and had open wounds, all of which posed a serious risk of infection. If left untreated, these injuries could have led to severe complications such as nerve damage, septicemia, or even death. Considering the severity of the condition, removing the affected tail was the most appropriate and life-saving option.

Neonatal traumatic injuries in non-human primates are infrequently reported and are often linked to maternal rejection, conspecific aggression, accidental trauma, or infanticide. While previous reports have described perineal injuries, bite wounds, and musculoskeletal trauma, severe sacro-coccygeal disruption requiring both caudectomy and anorectal reconstruction appears to be uncommon. To the best of our knowledge, this case represents one of the few documented descriptions of successful caudectomy combined with anorectal reconstruction in a neonatal non-human primate. This case therefore adds to the limited literature by highlighting the successful emergency management and reconstructive approach for a complex anorectal injury in a neonatal non-human primate.

Equally important was the decision to hand-rearing the neonate. Given the risk of repeated aggression, reintroduction into the group was not a safe option. Careful hand-rearing, along with structured feeding, thermal support, and continuous monitoring, played a crucial role in the infant's recovery and steady growth. These measures align with established principles of neonatal care, where maintaining body temperature, preventing infection, and ensuring adequate nutrition are key to survival.

Published growth references for neonatal Hamadryas baboons are scarce, limiting direct comparison of post-operative growth and development. Therefore, the observed growth trajectory was interpreted in the context of available morphometric data from baboons and other non-human primates. The progressive weight gain recorded during follow-up suggests that the nutritional regimen supported adequate growth and recovery. Nevertheless, the establishment of standardized growth benchmarks for neonatal Hamadryas baboons would improve the objective assessment of rehabilitation success and long-term health outcomes in future cases.

Overall, this case highlights the importance of vigilance during the periparturient period in captive primate management. It also shows that with timely rescue, appropriate surgical intervention, and dedicated care, even severe neonatal trauma can have a positive outcome. Beyond the clinical success, this case reinforces the need to combine behavioral understanding with veterinary preparedness to better manage and prevent such challenging situations.

## Conclusion

This report highlights that infanticidal or aggressive behavior can arise suddenly and without warning in captive Hamadryas baboon colonies, even among groups that appear socially stable. It underscores the importance of heightened vigilance and careful management during the period around birth, when infants are most vulnerable. The case also demonstrates that timely and well-executed surgical intervention can be life-saving in neonatal trauma, offering a meaningful chance for recovery and improved quality of life.

## Data Availability

The original contributions presented in the study are included in the article/supplementary material, further inquiries can be directed to the corresponding author.

## Footnotes

1 Milk solids, Demineralized whey, Soyabean oil, Corn oil, Minerals, Antioxidant (Soya lecithin), Oil mix (Arachidonic acid (ARA) from *Mortierella alpina* oil, Docosahexaenoic acid (DHA) from *Crypthecodinium cohnii* oil, High oleic sunflower oil, Antioxidants (Mixed Tocopherol Concentrate and L-Ascorbyl Palmitate) and Acidity regulator (Citric acid)), Vitamins, Taurin, Nucleotides, Acidity regulators (Citric acid and Potassium hydroxide) and L- Carnitine. INGREDIENTS AFTER RECONSTITUTION: Water, Milk solids, Demineralized whey, Soyabean oil, Corn oil, Minerals, Antioxidant (Soya lecithin), Oil mix (Arachidonic acid (ARA) from *Mortierella alpina* oil, Docosahexaenoic acid (DHA) from *Crypthecodinium cohnii* oil, High oleic sunflower oil, Antioxidants (Mixed Tocopherol Concentrate and LAscorbyl Palmitate) and Acidity regulator (Citric acid)), Vitamins, Taurin, Nucleotides, Acidity regulators (Citric acid and Potassium hydroxide) and LCarnitine.
